# Impact of universal mass vaccination with monovalent inactivated hepatitis A vaccines – A systematic review

**DOI:** 10.1080/21645515.2016.1242539

**Published:** 2016-10-27

**Authors:** Anke L. Stuurman, Cinzia Marano, Eveline M. Bunge, Laurence De Moerlooze, Daniel Shouval

**Affiliations:** a Pallas, Health Research and Consultancy BV, Rotterdam, The Netherlands; b GSK Vaccines, Wavre, Belgium; c Hadassah Hebrew University Hospital, Liver Unit, Jerusalem, Israel

**Keywords:** hepatitis A vaccine, incidence, long-term persistence, systematic review, universal vaccination

## Abstract

The WHO recommends integration of universal mass vaccination (UMV) against hepatitis A virus (HAV) in national immunization schedules for children aged ≥1 year, if justified on the basis of acute HAV incidence, declining endemicity from high to intermediate and cost-effectiveness. This recommendation has been implemented in several countries. Our aim was to assess the impact of UMV using monovalent inactivated hepatitis A vaccines on incidence and persistence of anti-HAV (IgG) antibodies in pediatric populations. We conducted a systematic review of literature published between 2000 and 2015 in PubMed, Cochrane Library, LILACS, IBECS identifying a total of 27 studies (Argentina, Belgium, China, Greece, Israel, Panama, the United States and Uruguay). All except one study showed a marked decline in the incidence of hepatitis A post introduction of UMV. The incidence in non-vaccinated age groups decreased as well, suggesting herd immunity but also rising susceptibility. Long-term anti-HAV antibody persistence was documented up to 17 y after a 2-dose primary vaccination. In conclusion, introduction of UMV in countries with intermediate endemicity for HAV infection led to a considerable decrease in the incidence of hepatitis A in vaccinated and in non-vaccinated age groups alike.

## Introduction

Most data on the incidence of acute HAV infection and prevalence of immunity cited in the literature are relatively old. According to World Health Organization (WHO) estimates, there were 126 million cases of acute hepatitis A in 2005.^1,2^ Acute hepatitis A-related morbidity and mortality increase with age. In children aged <6 years, ∼70% of infections are asymptomatic; if illness does occur, it is typically anicteric. In contrast, in older children, adolescents and adults, infection often leads to clinically overt acute hepatitis.^3,4^ Acute hepatitis A in adults may lead to prolonged incapacitation and rarely also to acute liver failure in previously healthy individuals and in patients with chronic liver disease.^5^ There is no specific treatment for acute hepatitis A except for supportive care and liver transplantation in the rare cases with liver failure.^6^

The virus is transmitted through the fecal-oral route, either through person-to-person contact or through contaminated food or water.^6^ The highest rates of infection are found in areas with poor sanitary conditions and hygienic practices and lack of access to clean water.^7,8^ Other risk factors for acquiring HAV include intravenous drug abuse and men having sex with men (MSM).^9^ Improvements in sanitation and access to clean water reduce viral circulation and infection and therefore the risk of waterborne HAV transmission and the overall rates of transmission. This reduction can be observed in the absence of vaccination as well as when vaccination programs are in place.

The first commercially produced hepatitis A vaccine was launched in 1992.^10^ Both inactivated and live attenuated vaccines against hepatitis A are currently available.^11^ A live attenuated vaccine is mainly used in China; most other countries use inactivated vaccines.^12^ Several monovalent inactivated hepatitis A vaccines are available, which are licensed for children aged one year or older (Table S1).^11-14^ The WHO considers that HAV vaccines of different brand names are interchangeable.^11^ The antigen content differs between vaccines,^14,15^ however, all are considered safe and immunogenic.^13,16-20^ Long-term persistence of antibodies has been shown with 2-dose vaccination schedules in adults.^21,22^

Areas with high viral transmission rates have a lower rate of severe morbidity and mortality than areas with lower viral transmission rates, as there are few susceptible adults in areas with high transmission rates.^2,23^ However, epidemiologic shifts from high to intermediate levels of HAV circulation, resulting from improvements in sanitation and hygiene, are paradoxically associated with an increase in susceptibility to infection due to decreasing immunity in the population as well as to more symptomatic disease due to older age at first infection.^7,10^ The impact of vaccination can therefore be confirmed by a decline of reported symptomatic cases, of fulminant hepatitis cases and of liver transplants.^24^ In these settings, the WHO recommends the integration of HAV vaccination into the national immunisation schedule for children aged one year and above, if indicated on the basis of incidence of acute hepatitis and consideration of cost-effectiveness.^1^ Most countries that have introduced hepatitis A vaccination in their immunisation programs use the available monovalent vaccines. Combined vaccines that include hepatitis A and B or hepatitis A and typhoid have also been developed. However, with the exception of Quebec in Canada^25^ and Catalonia in Spain^26^ where the combined hepatitis A and B vaccine is used in the, pediatric immunisation programmes, these are mainly intended for use in adult travelers or patients with specific risks like chronic liver diseases.^27^ Furthermore, hepatitis B vaccination has been introduced as a birth dose, monovalent or combined with other antigens, since the late 1990s or early 2000s in most countries. This review is therefore focused on the use of monovalent hepatitis A vaccine in the universal mass vaccination (UMV) setting.

Single-dose inactivated hepatitis A vaccines have been introduced in the national immunisation program in Argentina and additional countries in Latin America are considering adopting a similar protocol. This option seems to be comparable in terms of short and intermediate-term effectiveness, and is less expensive and easier to implement than the classical 2-dose schedule.^1,24^ However, until further long-term experience has been obtained with a single-dose schedule, in individuals at substantial risk of contracting hepatitis A, and in immunocompromised individuals, a 2-dose schedule may be preferable.^1^ Following an increase in the number of HAV outbreaks in the 1990s, Israel was the first country to introduce nationwide UMV for 18 months old toddlers using 2 doses of Havrix™.^28^ Additional countries that introduced UMV programs for hepatitis A include among others Argentina,^24^ Bahrain,^29^ Brazil,^30^ China,^31^ Greece,^32,33^ Panama,^34^ the US^35^ and Uruguay^36^; as well as regions of Belarus (Minsk City),^37^ Canada (Quebec),^25^ Italy (Puglia)^38^ and Spain (Catalonia).^39^

The objectives of this systematic review were to: (1) summarize data on the impact of monovalent inactivated hepatitis A vaccines in the context of UMV on the incidence of acute hepatitis A; (2) assess the impact of UMV on other parameters than incidence (e.g. indirect effects such as herd immunity); (3) summarize data on the long-term persistence of anti-HAV (IgG) antibodies in pediatric populations.

## Methods

### Search strategy

The PubMed, Cochrane, LILACS and IBECS databases were searched for literature in English, Spanish and Portuguese published between January 1^st^ 2000 and July 25^th^ 2016 (date of search). LILACS and IBECS are bibliographic databases with health science literature from Latin America, the Caribbean (LILACS) and Spain (IBECS). A search string combining terms on hepatitis A, vaccines and antibodies was built and adapted for use in each database. The search string used in PubMed was: “Hepatitis A Antibodies”[Mesh] OR “Hepatitis A Vaccines”[Mesh] OR “Hepatitis A/prevention and control”[Mesh] OR ((“Hepatitis A”[Mesh] OR “Hepatitis A virus”[Mesh] OR hepatitis A[tiab]) AND (“Vaccination”[Mesh] OR “Antibodies”[Mesh] OR vaccin*[tiab] OR immuniz*[tiab] OR immunis*[tiab] OR immune[tiab] OR immunity[tiab] OR immunology[tiab] OR antibod*[tiab])) OR Anti-HAV[tiab].

### Inclusion and exclusion criteria

In this systematic review only peer-reviewed primary research articles were included; review articles were excluded, but the reference lists of systematic reviews were screened to identify additional relevant primary articles. Review of the gray literature was not included. For review objectives 1 and 2, only observational studies conducted in a setting with UMV with monovalent, inactivated hepatitis A vaccines were included (Table S1). Studies from settings where hepatitis A vaccination was only implemented at the regional level (for example in Puglia, Italy^38^ or Minsk City, Belarus^37^), or from settings in which live attenuated hepatitis A vaccines or only combined hepatitis A vaccines were used in the UMV programs were excluded. Furthermore, studies in at risk populations, outbreak studies, modeling studies and economic evaluations were excluded; as were studies that did not present incidence or prevalence baseline data (i.e. data from the era prior to the introduction of UMV). For review objective 3, studies were only included if they were conducted with monovalent, inactivated hepatitis A vaccines in children (at time of primary vaccination) and provided follow-up data for a minimum of 5 y.

### Selection process

Articles were selected in 3 steps. Firstly, titles and abstracts identified through the search strategy were screened to identify potentially relevant articles. All titles and abstracts were screened in duplicate by 2 independent researchers. Any disagreements were resolved by the 2 reviewers by discussing the title and abstract; in case any doubts remained, the full-text was screened to ascertain if the article answered one of the research questions. Secondly, the full-text of the selected articles was screened, keeping in mind the inclusion and exclusion criteria described above, to determine whether it answered one of the review questions. If any aspects of the methodology were unclear, a comment was placed in the results table. Thirdly, for articles that presented duplicate data, the article that presented the most complete data (e.g., longer follow-up) was included.

## Results

**Figure 1.**
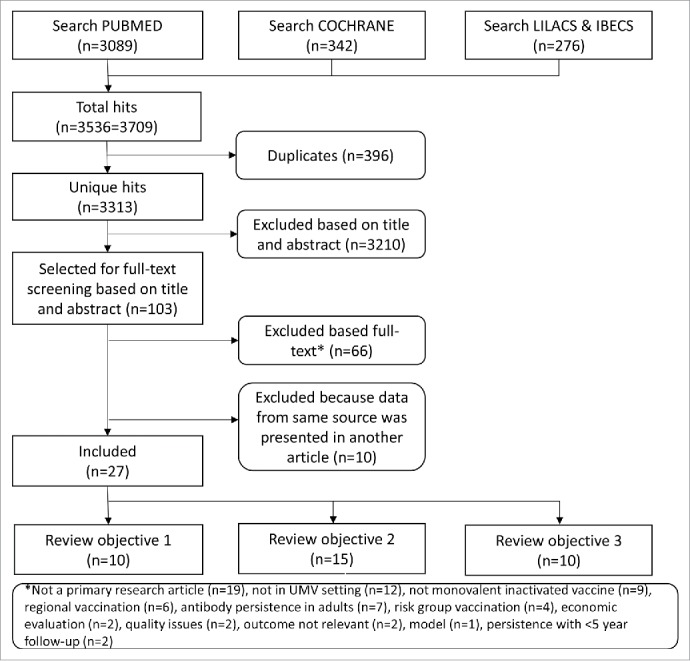
Flowchart of the selection procedure.

The search resulted in 3313 unique hits, of which 27 were included in this systematic review (Fig. 1). In total, 10 articles were included for review objective 1, 15 for review objective 2 and 10 for review objective 3. Some articles presented data for more than one review objective.

### Objective 1: Impact of UMV on HA incidence

#### Reduction in incidence

Ten studies provided data on incidence of acute hepatitis A before and after the introduction of hepatitis A UMV programs; all but one study (in Greece) found a marked decrease in acute hepatitis A incidence after UMV was implemented (Table 1). Declines were independent of the brand of the hepatitis A vaccine used in the programs; the number of doses that was given; the target age at first vaccination, which ranged from 12 to 24 months; or the attained vaccination coverage (range 25%–96.8%). After the introduction of UMV, the percent reduction in the incidence of acute hepatitis A was 88% in Argentina, >95% in Israel, 93% in Panama and 96% in Uruguay going form incidence rates ranging 6.0 to 142.4 per 100,000 population before vaccine introduction to a range of 0.4 to 7.9 per 100,000 population.

**Table 1.** Effect of universal mass vaccination programs for hepatitis A on incidence of acute hepatitis A.

	Vaccination							
Author; country	Start UMV; Target age	Vaccine coverage	Vaccine	Data source of hepatitis A cases	Years compared (before vs with UMV)^a^	Incidence (95%CI) per 100,000 population	Decline (%)	Comments^b^
Single-dose immunization strategy								
Vizzotti et al. 2013^27^ Argentina	2005 1 yr	96.8% for 2006–2011	Havrix˜ 720EU, Vaqta™ 25U, Avaxim˜ 80U, Virohep-A Junior 12UI	Early Alert Module, National Health Surveillance System; passive clinical surveillance	2000–2002 vs. 2006–2011	66.5 vs. 7.9	88.1	– –Source and year of denominator data n.r. – –COI: none
Two-dose immunization strategy								
Mellou et al. 2014^40^ Greece	2008 >12 mo	VC with 1 dose among 3 yr old children 80%, with 2 doses 42% in 2013	n.r	National surveillance data	2007 vs. 2013	2–3 vs. 2^c^	–^c^	COI: none
Chodick et al. 2008^64^ Israel	1999 18 mo	Received at least 1 dose:-<5 yrs: 9% (1999), 89% (2007)-5–14 yrs: 15% (1999), 68% (2007)	Havrix˜ 720EU^28^	Maccabi Healthcare Services; health maintenance organization	1998 vs. 2007	142.4 vs. 7.6^d^	95.0	– –MHS not representative of the general population (higher socio-economic class) (Daniel Shouval, personal communication, February 23, 2015) – –COI: none
Levine et al. 2015^47^ Israel	1999 18 mo	VC for vaccines given in 2003–2010 : 92% (dose 1), 88% (dose 2)	Havrix˜ 720EU	Cases reported to the national surveillance system	1993–1998 vs. 2008–2012	50.4 (35.9–64.9) vs. < 1.0	>98	COI: none
Estripeaut et al. 2015^50^ Panama	2007 > 12 mo	VC 1 dose 70.7% and 1 dose 40.1% in 2010	Havrix˜ 720EU	National surveillance systems	2000–2006 vs. 2010	51.1 vs. 3.7	93^e^	GlaxoSmithKline Biologicals SA
Romero et al. 2012^36^ Uruguay	2007 vaccination of children 1–5 yrs old In 2008 >12 mo	VC 1 dose 74%	n.r.	National surveillance system	2005 vs. 2010	69.6 vs. 2.7	96	COI: n.r.
Erhart et al. 2012^49^ Arizona, US	1999^c^ 2 yrs (1999) 1 yr (2006)	VC with 1 dose (by age): −24–59 mo: 36% (2000), 65% (2006) −5–9 yrs: 24% (2000), 77% (2006)	Havrix˜ 720EU, Vaqta˜ 25U^69^	Arizona Department of Health Services and local public health departments; passive surveillance	1994–1995 vs. 2006–2007	41 (41–42) vs. 2.6 (2.5–2.7)	94^e^	– –Year of denominator data n.r – –COI: n.r.
Ly et al. 2015^45^ US	1999^f^ (vaccinating states^g^) 2006 (all) 2 yrs (1999) 1 yr (2006)	n.r.	Havrix˜ 720EU, Vaqta˜ 25U^69^	National Notifiable Disease Surveillance System	1999 vs. 2011	6.0 vs. 0.4	93^e^	COI: none
Singleton et al. 2010^48^ Alaska, US	1999^f^ 2 yrs (1999) 1 yr (2006)	VC with ≥1 dose among children aged 24–35 mo:-2003: 72.7% (±95%CI 7.4)-2004: 69.9% (±95%CI 7.9)-2005: 66.8% (±95%CI 9.1)-2006: 65.9% (95%CI 57.1–73.7)	Havrix˜ 720EU, Vaqta˜ 25U^69^	Alaska Section of Epidemiology; surveillance	1994–1995 vs. 2002–2007	22.2 vs. 0.9	95.9^e^	COI: n.r.
Wasley et al. 2005^35^ Vaccinating states^g^, US	1999^f^ 2 yrs	VC for 1^st^ dose among children aged 24–35 mo in 2003, in states in which routine vaccination was: – Recommended: 50% – To be considered: 25%	Havrix˜ 720EU, Vaqta˜ 25U^69^	National Notifiable Diseases Surveillance System; passive national surveillance	1990–1997 vs. 2003	10.7 vs. 2.6	76	COI: none

CI: confidence interval; COI: conflict of interest; EU: ELISA units; HA: hepatitis A; MHS: Maccabi Healthcare Services; mo: months; n.r.: not reported; U: antigen units; UI: international unit; UMV: universal mass vaccination; US: United States; VC: vaccine coverage; yr(s): year(s)

a Years compared as available in the respective article. If more options were available, the most recent pre-vaccination and the most recent post-introduction year were used.

b Only industry-related conflicts of interest, funding source or financial disclosures reported

c Numbers read from graph. The incidence dropped to almost 0 in 2011 and rose again after that. Numbers read from graph therefore too imprecise to calculate % decline.

d Per 100,000 MHS members

e Calculated based using the formula [1-(HA incidence with UMV/HA incidence before UMV)]*100%

f Vaccination of American Indian and Alaska Native children started in 1996

g Vaccinating states: states in which HA childhood vaccination was recommend by the Advisory Committee on Immunization Practices (Alaska, Arizona, California, Idaho, Nevada, New Mexico, Oklahoma, Oregon, South Dakota, Utah, Washington) or considered (Arkansas, Colorado, Missouri, Montana, Texas, Wyoming) as of 1999

In Greece, a UMV program was initiated in 2008 however due the low endemcity level (<3.0 per 100,000 population) registered since the late 1980s, the program has not had significant impact on the notification rate of acute hepatitis A cases.^40^

### Objective 2: Impact of UMV on other measures and indirect effects

#### Impact on other outcomes

In Argentina between 2000–2003 approximately 17 cases of fulminant hepatitis A were reported while between 2008–2011, 2 y after the introduction of UMV, no more cases were reported (Table 2).^24^ A study that looked at hepatitis A outbreaks in day care centers in the Southern District of Israel showed that no more outbreak-related acute hepatitis A cases were reported.^41^ Hepatitis A vaccination was implemented is some US States as of 1999, the rate of hepatitis A-related ambulatory healthcare visits among enrollees going from 20.9 in 1996–1997 to 8.7 in 2004.^42^ The age-adjusted hepatitis A-mortality rate decreased significantly from 0.51 in 1999–1995 to 0.28 in 2000–2004.^43^ The 2011 hepatitis A incidence rate was the lowest ever recorded for the United States, data form the National Inpatient Survey have shown a reduction in the HA hospitalization rates from 0.64 in 2004–2005 vs. 0.29 in 2010–2011,^44^ however the relative rates of hospitalized hepatitis A cases among overall acute hepatitis A cases increased. In Greece, the number of HA-related hospital admission per 1000 hospital admissions among children dropped from 77.3 (95% CI 58.7–95.9) in 1999 (year of introduction of vaccine in private market) to 18.5 (95% CI 8.2–28.9) in 2013.^45^ Furthermore the outbreaks in 2013 among Roma populations did not spread to the general population.^40^

#### Indirect effects

A decline in acute hepatitis A incidence was seen in all age groups after the introduction of UMV in Israel in 1999^28,46,47^ as well as in the US, where vaccination was introduced in 1999 in some States,^35,48,49^ Such a drop was also recorded in Argentina in 2005^24^ and in Panama in 2007^50^ (Table 3).

**Table 2.** Effect of universal mass vaccination programs for hepatitis A on other outcome measures.

	Vaccination								
Author; country	Start UMV; Target age	Vaccine coverage	Brand, EU, doses	Data source	Outcome	Years compared	Before UMV	With UMV	Comments^a^
Single-dose immunization strategy									
Vizzotti et al. 2013^27^ Argentina	2005 1 yr	96.8% for 2006–2011	Havrix˜ 720EU, Vaqta˜ 25U, Avaxim˜ 80U, Virohep-A-Junior 12UI	4 pediatric centers in Buenos Aires and Unique Central National Institute of Ablation and Implant	Number of cases of HA-associated fulminant hepatic failure cases per year	2000–2003 vs. 2008–2011	∼17.5 ^b^	0	– No case definition of fulminant hepatic failure – COI: none
Two-dose immunization strategy									
Papaevangelou et al. 2016 [ref] Athens, Greece	2008 1 yr	88% (dose 1), 82% (dose 2) among children aged 6 yrs in 2012	n.r.	Infectious Disease Unit of a Tertiary Pediatric Hospital	HA hospital admission per 1000 admission among children aged 0–14 yrs	1999–2008 vs. 2009–2013	50.5 (95%CI 29.2–67.1)	20.8 (95%CI 19.2–30.1)	–COI: none
Belmaker et al. 2007^41^ Negev, Israel	1999 18 mo	Birth cohort 2000, by age 3 yrs: 86.4% (dose 1), 77.3% (dose 2)	Havrix˜ 720EU	Treating physicians, medical laboratories, Ministry of Education, concerned parents	Yearly average of HA cases associated with outbreaks in day care and school settings^c^	1993–1999 vs. 2001–2005	45.6	0	– Not clear how complete the notifications of outbreaks are (e.g. Ministry of Health not included as a source) – COI: n.r.
Collier et al. 2014^44^ US	1999^e^ (vaccinating states^f^) 2006 (nationwide) 2 yrs (1999) 1 yr (2006)^43^	n.r.	Havrix˜ 720EU, Vaqta˜ 25U^69^	National Inpatient Survey discharge data (∼20% of all community hospital discharges)	Hospitalizations for HA illness as principal diagnosis per 100,000 persons^d^	2004–2005 vs. 2010–2011	0.64	0.29	– Source and year of denominator data n.r. – COI: none
Ly et al. 2014^45^ US	1999^e^ (vaccinating states^f^) 2006 (nationwide) 2 yrs (1999) 1 yr (2006)	n.r.	n.r	National Notifiable Disease Surveillance System and Multiple Cause of death	% of HA-cases that was hospitalized; and HA-related mortality per 100,000 population^g^	1999 vs. 2011	7.3; 0.1	24.5; 0.02	COI: none
Vogt et al. 2008^43^ Vaccinating states^f^, US	1999 2 yrs	n.r.	Havrix˜ 720EU, Vaqta˜ 25U^69^	CDC's National Center for Health Statistics	Age-adjusted HA mortality rate per 1 million persons^h^	1990–1995 vs. 2000–2004	0.51	0.28	– –Denominator data from 2000–2004, but incidence data from 1990–2004 – –Some decline in mortality attributable to temporal trends, as reflected by statistically significant declines in mortality in states in which no large-scale vaccination programs were implemented – –COI: none
Zhou et al. 2007^42^ Vaccinating states^f^, US	1999 2 yrs	2004, children aged 2 yrs: 30.0%(in-plan vaccination rate)	Havrix˜ 720EU, Vaqta˜ 25U^69^	Medstat MarketScan database	HA-related ambulatory visits per 100,000 MarketScan enrollees	1996–1997 vs. 2004	20.9	8.7	– –Causal role for the vaccination program in decline is supported by greater decline in rate of HA ambulatory visits in vaccinating states vs. non-vaccinating states. – –Population somewhat homogeneous and not nationally representative. – –COI: n.r.

CDC: Centers for Disease Control and Prevention; CI: confidence interval; COI: conflict of interest; EU: ELISA units; HA: hepatitis A; NIS: National Inpatient Survey; n.r.: not reported; U: antigen units; UI: international unit; UMV: universal mass vaccination; US: United States; yr(s): years

a Only industry-related conflicts of interest, funding source or financial disclosures reported

b Outbreak in 2004, 27 cases of HA-associated fulminant hepatitis failure

c Outbreak: 2 or more reported cases of HA illness occurring within a month of each other in a day care facility, kindergarten, primary school or junior high school

d Extrapolated to nationwide hospitalizations using discharge weights

e Vaccination of American Indian and Alaska Native children started in 1996

f Vaccinating states: states with hepatitis A vaccination recommendations by the Advisory Committee on Immunization Practices (Alaska, Arizona, California, Idaho, Nevada, New Mexico, Oklahoma, Oregon, South Dakota, Utah, Washington) or considered (Arkansas, Colorado, Missouri, Montana, Texas, Wyoming) as of 1999

g HA listed as any cause of death in record axis, of HIV as underlying cause of death and HA as any other cause in record or entity axis

h HA listed as underlying cause of death

**Table 3.** Decline (%) in hepatitis A incidence by age group (years) before and after the introduction of universal vaccination.^a^

			Decline in hepatitis A incidence (%, with p-value or 95%CI when available)			
Reference; Country	Years compared (before UMV vs. with UMV)	Target age at 1st dose	Age groups with children younger than target age UMV program	Age groups with most vaccinated children	Other age groups	Oldest age groups
Vizzotti et al. 2014^27^ Argentina	2000–2002 vs. 2006–2011	1 yr	Age < 1: n.r.	Age 0–4: 90.5% (p < 0.0001) Age 5–9: 89.1% (p = 0.0004)	Age 10–14: 86.6% (p < 0.0001) Age 15–44: 72.8% (p < 0.0019)	Age > 45: 58.1% (p = 0.0033)
Dagan et al. 2005^28^ Israel	1993–1998 vs 2002–2004	1 yr	Age < 1: 84.3% (p < 0.005)	Age 1–4: 98.2% (p < 0.001) Age 5–9: 96.5% (p < 0.001)	Age 10–14: 95.2% (p = 0.01) Age 15–44: 91.3% (p < 0.001)	Age 45–64: 90.6% (p = 0.15) Age ≥65: 77.3% (p = 0.009)
Estripeaut et al. 2015^50^ Panama	2000–2006 vs.2010	12–18 mo	Age < 1: 100.0%	Age 1–4: 95.1	Age 5–9: 97.8% Age 10–14: 96.6% Age 15–19:91.7% Age 20–24:90.2% Age 25–49: 88.9%	Age ≥50: 61.8%
Ly et al. 2015^45^ All states, US	1999 vs. 2011	1 yr	Age <1: n.r.	Age 0–19: 95.9%	—	Age 20–39: 93.1%
Wasley et al. 2005^35^ Vaccinating states^b^, US	1990–1997 vs. 2003	2 yrs	Age < 2: 91.4% (95%CI 86.3–94.8)	Age 2–9: 95.6% (95%CI 94.8–96.1%) Age 10–18: 90.6% (95%CI 89.4–91.5%)	—	Age ≥19: 84.5% (95%CI 84.0–85.5)

CI: confidence interval; mo: months; UMV: universal mass vaccination; yr(s): year(s); n.r.: not reported; mo: months

a Age-specific groups as reported in the articles. Only studies with age-specific data in the main text or tables (rather than graphs) are shown.

b Vaccinating states: states in which HA childhood vaccination was recommend by the Advisory Committee on Immunization Practices (Alaska, Arizona, California, Idaho, Nevada, New Mexico, Oklahoma, Oregon, South Dakota, Utah, Washington) or considered (Arkansas, Colorado, Missouri, Montana, Texas, Wyoming) as of 1999

Vizotti et al describes a 1-dose vaccination program while the other publications refer to 2-dose vaccination programs.

Declines in incidence were generally highest in the age groups that contained the most vaccinated children.^24,28,35,51^ Incidence rates also dropped among children too young to be vaccinated in the programs.^28,35,50^ In most studies, the smallest declines in acute hepatitis A incidence were noted in the oldest investigated age groups.^24,28,35,50^ Similarly, a drop in hepatitis A-associated hospitalization rates was observed in non-vaccinated age groups in the US.^44^ In settings where many adults are likely to have natural immunity from prior infection, the drop in incidence in age groups not targeted by the UMV programs suggest a remarkable degree of herd immunity.

### Objective 3: Long-term persistence of anti-HAV antibodies

Of the 10 included studies that reported on persistence of anti-HAV (IgG) antibodies more than 5 y after vaccination of a pediatric population, 2 studies were performed in Argentina, one in Belgium, 2 in China, one in Israel and 4 in the US (Table 4). In six studies, authors reported that children who received booster vaccinations after the primary immunisation schedule were excluded from the follow-up analyses. Follow-up among the included studies ranged from 5 to 17 y. In the study with the longest follow-up, 87 to 100% (depending on the vaccination schedule) of the children whose antibody levels were measured at follow-up were found to be seroprotected up to 17 y after vaccination.^52^

**Table 4.** Long-term persistence of anti-hepatitis A antibodies.

Author Country	Intervention	Population	Number of participants	Outcome^a^: % (95%CI) seroprotected	Outcome^a^: GMC mIU/ml (95%CI)	Comments^b^
Espul et al. 2014^52^ Argentina	Avaxim˜ 80U Pediatric Gr1: 1 dose Gr2: 2 doses (schedule n.r.) Age at 1^st^ vacc: 11–23 mo	Healthy children. Ineligible for follow-up analyses if received additional dose of HA vaccine (Gr1: n=8; Gr2: n=1).	Gr1: 435 at 1 yr post-vaccination, 318 at 5 yr FU^c^ Gr2: 108 at 1 yr post-vaccination, 85 at 5 yr FU^c^	At 5 yr FU, %≥10 mIU/mL: Gr1: 99.7% (98.3–100.0) Gr2: 100.0% (95.8–100.0)	At 5 yr FU: Gr1: 122.5 (111.2–135.0) Gr2: 591.7 (479.9–729.4)	Subjects whose anti-HAV IgG titres fell <10 mIU/mL at any of the scheduled visits were offered a booster vaccination. Sanofi Pasteur.
Lopez et al. 2015^68^ Argentina	Avaxim˜ 80U Pediatric 2 doses (0, 6 mo)	Healthy children ≤ 15 years of age Ineligible for follow-up analyses if received additional dose of HA vaccine	54 at start, 30 at 14–15 yr FU^c^	At 14–15 yr FU, %≥20 mIU/mL 100.0%	At 14–15 yr FU: 253 (181–353)	Sanofi Pasteur
Van Herck et al. 2015^73^ Belgium	Gr 1: Epaxal˜ Junior Gr2: Epaxal˜ Gr3: Havrix˜ 720 EU 2 doses (0, 6 mo)	Healthy subjects	Gr1: 121 at start, 85 at 5.5 yr FU^d^ Gr2: 121 at start, 87 at 5.5 yr FU^d^ Gr3: 60 at start, 41 at 5.5 yr FU^d^	At 5.5 yr FU, %≥10 mIU/mL: 100% At 5.5 yr FU, %≥20 mIU/mL: Gr1: 98.8% Gr2: 100% Epaxal˜ Gr3: 97.6%	At 5.5 yr FU: Gr1: 171 (141–207) Gr2: 241 (201–287) Gr3: 152 (109–213)	Crucell Switzerland
Bian et al. 2010^74^ China	Havrix˜ 720 EU 2 doses (0, 6 mo) Mean age at 1^st^ vacc: 2.1 yrs	Children from Zhenhai District and Beilum District	200 at start, 110 at 10 yr FU^d^	At 10 yr FU, %≥5 mIU/mL: 99.09%	At 10 yr FU: 61.59 (51.92–73.07)	Children were “selected” to receive Havrix or live attenuated vaccine, but selection criteria n.r. COI: n.r.
Yu et al. 2016 [ref] China	Gr1: Healive 250 U Gr2: Havrix˜ 720 EU(randomly allocated) 2 doses (0, 6 mo)	Healthy children	Gr1: 300 at start, 230 at 5 yr FU^c^ Gr2: 100 at start, 79 at 5 yr FU^c^	At 5 yr FU, %≥20 mIU/mL: Gr1: 99.1% (96.9–99.9) Gr2: 97.5% (91.2–99.7)	At 5 yr FU: Gr1: 257.1 (226.9–291.4) Gr2: 168.1 (135.6–208.4)	COI: none
Dagan et al. 2016 [ref] Israel	Gr1: Epaxal˜ Jr (day 1) + RCV (day1) Gr2: Epaxal˜ Jr (day 1) + RCV (day 29) Gr3: Havrix˜ 720 EU (day 1) + RCV (day 1) (randomly allocated) 2 doses (0, 6 mo) Age at 1^st^ vac: 12–15 mo	Healthy children No HA vacc given during follow-up.	Gr1: 112 at start, 50 at 7.5 yr FU^d^ Gr2: 106 at start, 54 at 7.5 yr FU^e^ Gr3: 109 at start, 53 at 7.5 yr FU^d^	At 7.5 yr FU, % (95%CI) ≥10 mIU/mL: Gr1: 98.0% (89.4–99.9 Gr2: 96.3% (87.3–99.5) Gr3: 96.2% (87.0–99.5)	At 7.5 yr FU: Gr1: 85 (64–111) Gr2: 80 (61–105) Gr3: 61 (47–79)	Janssen Vaccines AG
Raczniak et al. 2013 (JID)^51^ US	Havrix˜ 360 EU^d^ 3 doses (schedule randomly allocated) Gr1: 0, 1, 2 mo Gr2: 0, 1, 6 mo Gr3: 0, 1, 12 mo Age at 1^st^ vacc: 3–6 yrs	Alaska Native children. Ineligible at 17 year follow-up if received a booster dose of HA vaccine (n=30)	Gr1: 49 at start, 23 at 17.3 yrs FU^d^ Gr2: 42 at start, 17 at 17.3 yrs FU^c^ Gr3: 45 at start, 18 at 17.3 yrs FU^c^	At 17.3 yr FU, %≥20 mIU/mL: Gr1: 87% Gr2: 100% Gr3: 94%	At 17.3 yr FU: Gr1: 129 (61–270) Gr2: 235 (125–445) Gr3: 354 (143–880)	COI: none
Raczniak et al. 2013 (Vaccine)^56^ US	Havrix˜ 720 EU or Vaqta˜ 720 EU 2 doses (0, 6–12 mo) Age at 1^st^ vacc: 1–4 yrs (50.5%), 5–9 yrs (31.7%), >10 yrs (17.8%)	Alaska Native children	101 at start, 57 at ≥11 yrs FU^d^	At ≥11 yrs FU, %≥20 mIU/mL: 95%	≥13 to <15 yrs FU, by age at first dose^f^: 1–2 yrs (n=5): 21 (6–77) 3–6 yrs (n=5): 80 (40–159) ≥7 yrs (n=1): 81	Participants were evaluated for follow-up only once (i.e. those evaluated before 11 years not counted in follow-up) COI: none
Spradling et al. 2016 [ref] US	Havrix˜ 720EU 2 doses (0 and 6 mo) Age at 1^st^ vacc (randomly allocated): Gr1: 6 mo Gr2: 12 mo Gr 3: 15 mo	Children who participated in immunogenicity study; enrolled from prenatal and pediatric clinics in Anchorage. Ineligible for long-term follow-up if received additional dose of HA vaccine (n=11)	Gr1: at start, 38 (20+, 18-) at 15–16 yrs of age Gr2: at start, 26 (17+, 9-) at 15–16 yrs of age Gr3: at start, 31 (19+, 12-) at 15–16 yrs of age	At 15–16 yrs of age, %>20 mIU/mL: Gr1-: 75% Gr1+: 61% Gr2-: 100% Gr+: 67% Gr3-: 100% Gr3+: 67%	At 15–16 yrs of age: Gr1-: 49 (31–77) Gr1+: 27 (16–44) Gr2-: 78 (49–123) Gr2+: 35 (14–87) Gr3-: 58 (37–90) Gr3+: 50 (24–103)	COI: none
Fiore et al. 2003^53^ US	Havrix˜ 360 EU^d^ 3 doses (0, 2, 4 mo) Age at 1^st^ vacc: 2 mo	Infants in immunogenicity trial who responded to primary series Children who had received additional doses of HA vaccine since the 1991 study excluded (n=n.r.)	78 at start, 48 (31-, 17+) at 6.1 yrs FU^g^	At 6.1 yrs FU, %≥33 mIU/mL: -: 68% +: 24%	At 6.1 yrs FU: -: 50 (31–81) +: 18 (10–32)	SmithKline Beecham Biological

CI: confidence interval; COI: conflict of interest; EU: ELISA units; FU: follow-up; Gr: group; HA: hepatitis A; HAV: hepatitis A virus; mo: months; n.r.: not reported; RCV: routine childhood vaccines; U: antigen units vacc: vaccination; yr(s): years;

a + and – refer to maternal anti-HAV antibody status

b Only industry-related COI, funding source or financial disclosures reported

c After first dose

d After second dose

e This formulation is no longer available

f ≥11 to <13 yrs FU, GMC by age at first dose: 1–2 yrs (n=26): 98 (66–147); 3–6 yrs (n=8): 298 (51–1749); ≥7 yrs (n=11): 211 (112–397). ≥15 years FU, age at first dose 3–6 yrs (n=1): 43 mIU/mL

g After third dose

The vaccination schedule, the number of doses, the antibody-status of the mother and age at vaccination were all found to influence the height of the geometric mean concentration (GMC) of anti-HAV antibodies. In the study with the longest follow-up, children who received the third dose of hepatitis A vaccine at month 12 compared to month 2 of the vaccination schedule had a higher GMC after 17 y (354 mIU/mL [95%CI 142–880] vs.129 mIU/mL [95%CI 61–270]).^52^ In another study, the GMC at 5 y follow-up was significantly higher among those who had received 2 doses of hepatitis A vaccine compared to those who had received only one dose (592 mIU/mL [95%CI 480–729] vs. 123 mIU/mL [95%CI 111–135]).^53^ In 2 studies, the presence of maternal antibodies was significantly associated with lower GMCs at 6 and 15 y follow-up among infants vaccinated at aged 2 and 6 months, respectively.^54,55^ One of these studies also showed that the GMC was higher among children vaccinated at age 12 or 18 months compared to those aged 6 months.^55^

## Discussion

In 2012, Ott et al. reviewed the literature on the long-term protective effect of hepatitis A vaccines,^56^ and new studies have been published since then.^52,53,55,57-59^ To our knowledge, the present communication is the first systematic review that examines the overall impact of universal mass vaccination with inactivated hepatitis A vaccines.

### Impact of UMV programs

The goal of the hepatitis A UMV programs in countries with intermediate endemicity for HAV is to protect individuals from infection and disease and reducing the virus circulation. Most UMV programs are aimed at very young children, as they represent the reservoir of the infection representing an important vehicle in the transmission of HAV.^28,60-63^ UMV programs are generally based on 2-dose vaccination, however Argentina and Brazil have decided to introduce a one dose only program.^24,30^ This review focused only on countrywide UMV with monovalent inactivated vaccines. In China, hepatitis A vaccination was introduced into the routine childhood program in 2008. However, as 92% of the 16 million children aged 18 months are vaccinated annually with a single dose of live attenuated vaccines, data from China was beyond the scope of this review.^64,65^ Likewise, some areas implemented vaccination only in a particular region of a country (e.g. Puglia, Italy^38^; or Catalonia, Spain where a combined hepatitis A and B vaccine was used^39^) or only in one city (e.g., Minsk, Belarus^37^), and were not included.

All but one of the reviewed studies that looked at incidence of acute hepatitis A showed a marked decline in the incidence after the introduction of hepatitis A UMV programs in countries with intermediate levels of endemicity defined elsewhere.^66^ However reductions in the rate of transmission are also attributable to the improved hygiene resulting from cleaned water access. It has been in fact shown that Hepatitis A virus (HAV) is associated with inadequate water and sanitation, the increases in clean water access lead to reduced risk of waterborne HAV transmission.^67^ The incidence in non-vaccinated age groups was also found to decrease, likely indicating a strong impact of vaccination programs on herd immunity.^24,28,35,46,48,49^ However an increase in the proportion of acute hepatitis A cases hospitalized in the United States was reported and this could be explained by the increase in age of the susceptible population which is predominantly adults more prone to clinically overt and severe disease.^44,51^

Greece is the only country where the introduction of UMV for hepatitis A did not show a strong impact on the incidence of hepatitis A, as reported in other countries. Differently than in other countries the UMV in Greece was implemented when already at least a third of children on a national level had been vaccinate in the private market and specifically data from Athens metropolitan area showed a vaccine uptake >50% prior to the UMV introduction. The low impact of UMV showed in Greece is likely due to the fact that Greece was already a country with low endemicity at the time UMV was introduced,^66^ and that children in the main high risk group, the Roma population, are not vaccinated at the recommended level to prevent transmission.^40^

The evaluation of the impact of hepatitis A vaccination has been carried out mostly using national or state-wide passive surveillance systems, based primarily on laboratory-confirmed or epidemiologically-linked cases of acute hepatitis A,^24,35,36,40, 47-50^ and 2 Israeli studies used health insurance organization data.^46,68^ None of the systems ascertained asymptomatic infections or those with acute hepatitis A disease that did not seek medical care. None of the authors reported any changes in underreporting over time. If these factors had any effect on the outcome, it is likely they led to an underestimation of the reduction in acute hepatitis A, rather than an overestimation.

Outbreaks have often been the direct trigger for the introduction of UMV programs, such as the 2003–2004 outbreaks in Argentina.^24^ In some areas, the overall incidence of acute hepatitis A was already declining in the decade prior to the introduction of routine vaccination programs, but maintained its cyclic pattern.^28,35,48^ For example, Singleton et al. state that increasing rates of in-home running water perhaps contributed to somewhat lower HAV incidence in the later pre-licensure period in Alaska.^48^ However, the decline in incidence after the introduction of UMV was unprecedented in magnitude. No major changes in water or sanitation infrastructure were reported that coincided with the introduction of UMV and to which the decrease could be attributed.^24,28,48^ Furthermore the decline can't be entirely explained by the cyclical nature of the disease, as the decline was accompanied by shifts in the relative age distribution of acute hepatitis A to older age groups^24,35,44,46,68^ and declines were larger in vaccinating than non-vaccinating states of the US.^35^ Finally, epidemic peaks have been disappearing. The decline in incidence was sustained over time; studies in this review included up to 8 y of data post-UMV introduction,^48,49,68^ during which no increase in acute hepatitis A incidence was observed.

Vaccination coverage varied widely among geographical areas; reaching over 95% of young children in Argentina, but only 25% in US states where vaccination was “considered” from 1999. Efforts to target children at highest risk in certain areas of the US might explain the high impact of UMV despite this low vaccination coverage.^35^ The impact of UMV on acute hepatitis A incidence was evident despite limited vaccine coverage rates in some countries.

Due to the heterogeneity of the data, meta-analysis was not performed. The periods compared in the before vs. after comparison differ too much between the studies, especially in terms of years since the introduction of UMV. Additionally, Vizzotti et al^24^ describes a 1-dose vaccination program while the other publications refer to 2-dose vaccination programs. As with any systematic literature review, this review is subject to the limitations of the included articles.

Almost all included studies showed a decline in the incidence following the introduction of hepatitis A UMV programs. As these studies were conducted in settings with intermediate endemicity, the results should be interpreted within this context and differences in the surveillance systems should also been considered when interpreting the data. In the study from Greece, a country with low endemicity, no such decline was seen. Improved hygiene over the past century has led to low endemicity in much of the developed world. The resulting high susceptibility may increase the risk of outbreaks when exposure does occur, and this has been the cause of recent large outbreaks through food contamination, e.g. through frozen pomegranate arils in the United States^69^ and frozen berries in Europe.^70^ However, this does not necessarily indicate that mass immunization programs with hepatitis A vaccine should be introduced in low endemic countries.

#### Long-term persistence

In this review, evidence of long-term persistence of anti-HAV (IgG) antibodies in children for up to 17 y following vaccination with monovalent inactivated vaccines was found.^52^ However, these data have been generated following vaccination with 3 doses of an inactivated Hepatitis A vaccine containing 360 EU per dose (HAVRIX˜ 360 EU), not used anymore. Long-term immune memory is important so that children vaccinated under childhood vaccination programs will still be protected by the time they reach the age at which disease is likely to be symptomatic.^12,71^ Infants vaccinated before the age of one year appear to have a lower antibody response.^54,55^ This supports the target age of one year or older for children immunized in UMV programs. A recent model-based assessment of vaccine induced immune memory against HAV in adults suggests that anti-HAV antibodies will persist for at least 20 y in >95% of vaccines.^22^

A limitation in interpretation of most long-term persistence studies is that a fraction of the children that received the primary vaccination series also received an additional booster dose before the last follow-up. Indeed, boosters complicate the interpretation of follow-up data. For instance, 5 of the included studies reported that children who received booster doses were excluded^52-55,72^; this results in an overestimation in the GMCs and the percentage of children who were seropositive years after the primary vaccination series, as boosters were likely given to precisely those children whose antibodies levels dropped below a certain threshold. The interpretation is further hampered by the fact that most studies did not report how many children were excluded for this reason. For these studies, it can be concluded that anti-HAV-antibodies can persist up to the time of last follow-up (6 to 14–15 years).^54,58,59,72-74^ For the 2 studies that did report how many children were excluded due to the receipt of a booster dose, it can be concluded that antibodies persist to the time of last follow-up (5 and 17 y) in the majority of children who were not lost to follow-up.^52,53,55,57^

A limitation of all studies on long-term persistence of antibodies and immune memory is the large number of participants that were lost to follow-up over time. Additionally, the possibility that the children received a ‘natural booster’ due to exposure to circulating HAV cannot be excluded, especially in the early years of the vaccination programs. However, there is also no proof that the long-term persistence of antibodies was the result of natural boosting.^54^ Furthermore, seroprotection against HAV was defined as a GMC of at least 5, 10, 20, or 33 mIU/mL depending on individual assays and vaccines used in the included studies. However, the true lowest limit of anti-HAV that confers protection is unknown and might be even lower than the detection limit of a particular assay.^71,75^

Information on long-term persistence after administration of a single dose of vaccine in children is limited, but has been documented in adults.^76-78^ This information would suggest that protective anti-HAV antibody levels after a single dose of inactivated hepatitis A vaccine can persist for up to 11 y. A recent publication also suggests that antibody titers are lower and antibodies decay faster in younger children (aged 1–7 years).^74^ Long-term persistence data after one dose in children would therefore be valuable, especially as not all children are vaccinated twice, either because the second dose is missed or because only one dose is recommended, e.g., in Argentina and Brazil.

The impact on the disease incidence and other related health outcomes as well as the long term antibody persistence provided by the vaccination are all critical considerations of vaccine program impact, however every country must also assess the cost-effectiveness of the program in deciding for or against the implementation of hepatitis A UMV. The data reported present certain heterogeneity in terms of epidemiology and reporting systems and therefore the data should not be read as a comparison of the impact of immunization programs in the different country but solely as a descriptive assessment of the country by country outcomes.

## Conclusion

Introduction of UMV with monovalent inactivated hepatitis A vaccines in countries with intermediate endemicity for HAV infection led to a considerable decrease in the incidence of hepatitis A in vaccinated and in non-vaccinated age groups alike.

## Supplementary Material

KHVI_A_1242539_Supplementary_material.zip

## Abbreviations

UMV: universal mass vaccination
HAV: hepatitis A virus
WHO: world health organization
MSM: men having sex with men
GMC: geometric mean concentration
