# Supplementary material for: Impact of universal mass vaccination with monovalent inactivated hepatitis A vaccines – A systematic review
Source: Hum Vaccin Immunother. 2016 Oct 27;13(3):724–36. doi: 10.1080/21645515.2016.1242539 (PMC5360128; doi:10.1080/21645515.2016.1242539)
Supplement: KHVI_A_1242539_Supplementary_material.zip [file khvi-13-03-1242539-s001.zip › KHVI_A_1242539 Supplementary material.docx]

**Supplemental Material**

**Table S1. Monovalent inactivated Hepatitis A vaccines***

|  | | **Vaccines** | | | | |
| --- | --- | --- | --- | --- | --- | --- |
|  |  | **HAVRIX**^TM^  **(GSK)** | **EPAXAL**^TM^  **(Crucell)** | **VAQTA**^TM^  **(Merck)** | **AVAXIM**^TM^  **(SP-MSD)** | **HEALIVE**^TM^  **(Sinovac Biotech)** |
| **Presentation** | **Adults** | Havrix^TM^ Monodose | Epaxal^TM^** | Vaqta^TM^ | Avaxim^TM^ | Healive^TM^ Adult |
|  | **Children** | Havrix^TM^ Junior Monodose | Epaxal^TM^ Junior | Vaqta^TM^  Paediatric | Avaxim^TM^ Pediatric | Healive^TM^ Pediatric |
| **Characteristics** | **Volume** | Adult: 1ml Paed: 0.5ml | Adult: 0.5ml Paed: 0.25ml | Adult: 1.0ml Paed: 0.5ml | Adult: 0.5ml Paed: 0.5ml | Adult: 1.0ml Paed: 0.5ml |
|  | **Antigen dosage** | Adult: 1440 EU Paed: 720 EU | Adult: ≥ 24IU Paed: ≥ 12IU | Adult: 50U Paed: 25U | Adult: 160U Paed: 80U | Adult: 500U Paed: 250U |
|  | **Year launched** | 1994 | 1994 | 1996 | 1996 | 2002 |
|  | **Antigen** | Hepatitis A virus Antigen  HM175 strain *cultured on MRC-5 human diploid cells* | Inactivated Hepatitis A Virion   RG-SB strain *cultured on MRC-5 human diploid cells* | Inactivated hepatitis-A  whole virus  Strain CR 326F *cultured on MRC-5 human diploid fibroblasts* | Inactivated hepatitis-A whole virus   GBM strain *cultured on MRC-5 human diploid cells* | Inactivated hepatitis-A whole virus  Strain TZ84 *cultured on human fetal lung diploid fibroblast 2BS cells* |
|  | **Vaccination schedule** | **2nd dose: 6-12 months** | **2nd dose: 6-12 months** | **2nd dose: 6-18 months** | **2nd dose: 6-12 months** | **2nd dose: 6 months** |

*Adapted from Shouval et al. 2011 [[11](file:///C:\\Users\\Anke\\Documents\\Pallas%20-%20Hepatitis%20A%20manuscript\\Submission%203\\Re-submission\\Havrix%20Lit%20Review_20160502_with%20tablesANDrefs.doc#_ENREF_11)]. **Also sold under the brand names HAVpur and VIROHEP-A

EU: ELISA units; U: antigen units; IU: international unit

All inactivated hepatitis A vaccines are deemed comparable and interchangeable by WHO (WHO position paper on hepatitis A vaccines –June 2012 No. 28-29, 2012, 87, 261–276 <http://www.who.int/wer>).
